# Effects of Rhythmic Auditory Cueing in Gait Rehabilitation for Multiple Sclerosis: A Mini Systematic Review and Meta-Analysis

**DOI:** 10.3389/fneur.2018.00386

**Published:** 2018-06-11

**Authors:** Shashank Ghai, Ishan Ghai

**Affiliations:** ^1^Institute of Sports Science, Leibniz University Hanover, Hanover, Germany; ^2^Victor Chang Cardiac Research Institute, Sydney, NSW, Australia

**Keywords:** rhythm perception, gait, movement disorders, rehabilitation, falls, spasticity

## Abstract

Rhythmic auditory cueing has been shown to enhance gait performance in several movement disorders. The “entrainment effect” generated by the stimulations can enhance auditory motor coupling and instigate plasticity. However, a consensus as to its influence over gait training among patients with multiple sclerosis is still warranted. A systematic review and meta-analysis was carried out to analyze the effects of rhythmic auditory cueing in studies gait performance in patients with multiple sclerosis. This systematic identification of published literature was performed according to PRISMA guidelines, from inception until Dec 2017, on online databases: Web of science, PEDro, EBSCO, MEDLINE, Cochrane, EMBASE, and PROQUEST. Studies were critically appraised using PEDro scale. Of 602 records, five studies (PEDro score: 5.7 ± 1.3) involving 188 participants (144 females/40 males) met our inclusion criteria. The meta-analysis revealed enhancements in spatiotemporal parameters of gait i.e., velocity (Hedge's g: 0.67), stride length (0.70), and cadence (1.0), and reduction in timed 25 feet walking test (−0.17). Underlying neurophysiological mechanisms, and clinical implications are discussed. This present review bridges the gaps in literature by suggesting application of rhythmic auditory cueing in conventional rehabilitation approaches to enhance gait performance in the multiple sclerosis community.

## Introduction

Multiple sclerosis is a prevalent, progressive demyelinating disease of the central nervous system (1). It is one of the most common causes of non-traumatic progressive disability in younger population groups (2, 3), but is also not uncommon in aged population (4). The main pathological characteristics of multiple sclerosis include progressive demyelination, and disruption of blood brain barrier due to inflammatory changes (5). This eventually affects the functioning of relevant axonal tracts, thereby causing widespread neurological symptoms (1, 6). The clinical manifestations in patients with multiple sclerosis include disruptions in sensory, motor and cognitive functioning. For instance, paresthesia, sensory loss, progressive hemiparesis, ataxia, fatigue, and depression have been widely reported (7, 8).

Gait and postural dysfunctions are also common in patients with multiple sclerosis especially due to the involvement of pyramidal track, cerebellar and spinal cord dysfunctions (9–11). Prosperini et al. (2) for instance, reported lesions primarily in cerebellar, supratentorial associative bundles to affect the static and dynamic stability in patients with multiple sclerosis. Likewise, pathological involvement of leukocortical, intracortical, and subpial regions have also been reported (7, 12). Together, these sensory, motor and cognitive dysfunctions affect motor control and coordination (13, 14), eventually promoting falls (15), and affecting the quality of life (16). Typical gait characteristics exhibited by patients with multiple sclerosis include reduced gait velocity, stride length, cadence, and increased step width, asymmetric gait, double limb support duration (17, 18) [for a detailed review see (16, 19)]. Kinematic analysis of gait further reports larger range of motion at hip joint (20), increased knee flexion, reducing in ankle plantarflexion (21), and higher pelvic obliquity (22). Furthermore, electromyographic studies report abnormal musculoskeletal co-activation pattern especially at the ankle joint (23). These adjustments in gait kinematics and muscular co-contractions have been affirmed as cautionary measures adopted by patients for promoting stability during gait (24). These gait modifications although are intended to safeguard oneself from falling. Retrospectively, these modifications promote a rather slow, uneconomical, fatigue promoting, and highly fall prone gait pattern (25–28).

Common treatment strategies to curb motor dysfunctions in multiple sclerosis include physical exercise (29, 30), training with virtual-reality (31), physical/occupational therapy (32), hydrotherapy (33), electrical stimulations (16), martial arts (34), dual-task training (28), and external sensory cueing (35, 36). Studies report that sensory dysfunctions in patients with multiple sclerosis primarily play a key role in disrupting motor control and coordination (37). Disruptions in the perception of visual (38), and proprioceptive (39, 40), systems have been well-documented. Therefore, providing additional sensory cueing to support movement execution might serve as a viable option to overcome this loss. Only a handful of studies have analyzed the effects of external sensory stimulations (auditory, visual) on motor performance in patients with multiple sclerosis (35, 36, 41, 42). Nevertheless, the predominant role of auditory cueing as compared to its visual counterpart has been emphasized in literature (43, 44). Predominantly auditory cortex has been reported to perceive rhythmic stimuli by as short as 20–30 ms, which is considerably shorter as compared to visual and tactile thresholds (45–47). Moreover, it utilizes the rich interconnectivity of the auditory cortex to motor centers from spinal cord extending from the brainstem, cortical and subcortical structures (48–50). This also enables the auditory system to operate in a quite fast, precise, and efficient manner (51, 52). Several types of rehabilitation approaches have been reported in the literature for delivering external auditory stimulations, such as rhythmic auditory cueing (50), patterned sensory enhancement (53, 54), and real-time auditory feedback (55, 56). However, rhythmic auditory cueing is the most widely studied treatment strategy with respect to healthy population groups (28), population groups, and patients affected from movement disorders such as parkinsonism (47), stroke (57), and cerebral palsy (58). This type of stimulation can allow enhancements in motor execution in a multifaceted manner (47, 52). For instance, the sensory cueing can enhance biological motion perception (55, 59), promote audio-motor imagery (60, 61), reducing shape variability in muscle co-activation (62), mediate cortical reorganization, neural-plasticity (63), reduce cognitive overload (64), and more (45).

Moreover, recent research suggests increased financial burden on patients with multiple sclerosis (65, 66), especially because of the disease's progressive and relapsing nature (67). Therefore, development of affordable, and convenient rehabilitation strategies must be emphasized. Rhythmic auditory cueing is an effective strategy in these terms as it is viable, cheap, and can also be effectively applied as a home-based intervention (26–28). Therefore, we attempted to develop a state of knowledge by conducting a systematic review and meta-analyses to determine the effects of rhythmic auditory cueing on gait performance in patients with multiple sclerosis.

## Methods

This review was conducted according to the guidelines outlined in Preferred Reporting Items for Systematic Reviews and Meta-analysis: The PRISMA statement (68).

### Data sources and search strategy

Academic databases such as Web of science, PEDro, EBSCO, MEDLINE, Cochrane, EMBASE, and PROQUEST were searched from inception until December 2017. A sample search strategy has been provided in (Table 1).

**Table 1:** Sample search strategy EMBASE.

**DATABSE**	**EMBASE**
**DATE**	**10/12/2017**
**STRATEGY**	**#1 AND #2 AND #3 AND #4 AND #5 AND #6 AND #7**
**#1**	(“rhythmic auditory cueing” OR “rhythmic auditory cueing” OR “rhythmic acoustic cueing” OR “rhythmic auditory entrainment” OR “metronome cueing” OR “metronome” OR “rhythmic metronome cueing” OR “acoustic stimulus” OR “acoustic cueing” OR “acoustic cueing” OR “external stimuli” OR “external cueing” OR “external cueing” OR “music therapy” OR “Neurological music therapy” OR “tempo” OR “beat” OR “rhythm” OR “RAC” OR “NMT” OR “real-time auditory cueing” OR “sonification”)/de OR (rhythmic auditory cueing OR rhythmic auditory cueing OR rhythmic acoustic cueing OR rhythmic auditory entrainment OR metronome cueing OR metronome OR rhythmic metronome cueing OR acoustic stimulus OR acoustic cueing OR acoustic cueing OR external stimuli OR external cueing OR external cueing OR music therapy OR Neurological music therapy OR tempo OR beat OR rhythm OR RAC OR NMT OR real-time auditory cueing OR sonification)ti,ab
**#2**	(“MS” OR “Multiple sclerosis” OR “Acute fulminating sclerosis” OR “disseminated sclerosis”)/de OR (MS OR Multiple sclerosis OR Acute fulminating sclerosis OR disseminated sclerosis))ti,ab
**#3**	(“walking” OR “gait” OR “locomotion” OR “range of motion” OR “ROM” OR “ambulation” OR “mobility” OR “treadmill gait” OR “balance” OR “stability” OR “stride” OR “gait training” OR “gait rehabilitation”)/de OR (walking OR gait OR locomotion OR range of motion OR ROM OR ambulation OR mobility OR treadmill gait OR balance OR stability OR stride OR gait training OR gait rehabilitation);ti,ab
**#4**	(“rehabilitation” OR “treatment” OR “rehab” OR “management” OR “therapy” OR “physiotherapy” OR “physical therapy” OR “prevention” OR “risk prevention”)/de OR (rehabilitation OR treatment OR rehab OR management OR therapy OR physiotherapy OR physical therapy OR prevention OR risk prevention);ti,ab
**#5**	(“age groups” OR “adolescent” OR “young” OR “elderly” OR old) AND (gender OR “male” OR “female”)/de OR [age groups OR adolescent OR young OR elderly OR old AND (gender OR male OR female)];ti;ab
**#6**	(“intervention study” OR “cohort analysis” OR “longitudinal study” OR “cluster analysis” OR “crossover trial” OR “cluster analysis” OR “randomized trial” OR “major clinical study”)/de OR (longitudinal OR cohort OR crossover trial OR cluster analysis OR randomized trial OR clinical trial OR controlled trial);ti,ab

### Data extraction

Upon selection for review, the following data were extracted from each article i.e., author, date of publication, selection criteria, sample size, sample description (gender, age, health status), intervention, characteristics of auditory cueing, outcome measures, results, and conclusions. The data were then summarized and tabulated (Table 2).

**Table 2:** Studies analyzing the effects of rhythmic auditory cueing on gait in patients with multiple sclerosis.

**Author**	**Research question(s)/hypothesis**	**Sample description, age: (M ±*SD*)**	**PEDro score**	**Assessment**	**Research design**	**Auditory signal characteristics**	**Conclusions**
Shahraki et al. (69)	Effects of auditory cueing on gait in patients affected from multiple sclerosis	Exp: 7F, 2M (40.3 ± 6.6) Ct: 7F, 2M (38.1 ± 12.1)	4	Stride length, stride time, double support time, cadence & gait velocity	Pre-test, gait training with rhythmic auditory cueing at +10% of preferred cadence for 30 min/session, 3 times/week for 3 weeks, post-test	Rhythmic metronome cueing at +10% of preferred cadence	Significant enhancement in stride length, gait speed, cadence in Exp as compared to Ct & after training with auditory cueing. Significant reduction in stride time & double support time after training with auditory cueing. Significantly reduced stride time in Exp as compared to Ct.
Seebacher et al. (70)	Effects of rhythmic auditory cueing and motor imagery on gait in patients affected from multiple sclerosis	Exp I: 25F, 9M (43.8) Exp II: 29F, 5M (45.4) Ct: 31F, 2M (43.1)	7	Timed 25-foot walk test, 6-min walk test, multiple sclerosis walking scale 12, modified fatigue impact scale, short-form 36 health survey, multiple sclerosis impact scale 29 & Euroquol 5D 3L questionnaire	Pre-test, motor imagery training (internal gait simulation with fast gait, wider steps…) with rhythmic auditory cueing for 17 min session, 6 times/week for 4 weeks, post-test	Rhythmic auditory cueing at preferred cadence Exp I: Instrumental music: cueing at 2/4, 4/4 meter, emphasis on 1st & 3rd beat. Exp II: metronome cueing at 2/4, 4/4 meter, emphasis on 1st & 3rd beat. Rhythmic verbal cues by researcher (heel off, toe off…)	Significant enhancement in 6-min walking distance in both Exp I & II after receiving auditory cueing, as compared to Ct. Significant reduction in timed 25-foot walking time, modified fatigue impact scale in both Exp I & II after receiving auditory cueing, as compared to Ct. However, Exp I had better benefits as compared to Exp II. Significant enhancement in short-form 36 health survey, multiple sclerosis impact scale 29 & Euroquol 5D 3L questionnaire i.e., quality of life, in both Exp I & II after receiving auditory cueing, as compared to Ct. However, Exp I had better benefits as compared to Exp II.
Seebacher et al. (71)	Effects of rhythmic auditory cueing and motor imagery on gait in patients affected from multiple sclerosis	Exp I: 10F (47.3) Exp II: 7F, 3M (41.8) Ct: 5F, 5M (46.1)	6	Timed 25-foot walk test, 6-min walk test, modified fatigue impact scale	Pre-test, motor imagery training (internal gait simulation with fast gait, wider steps…) with rhythmic auditory cueing for 17 min session, 6 times/week for 4 weeks, post-test	Rhythmic auditory cueing at preferred cadence Exp I: Instrumental music: cueing at 2/4, 4/4 meter, emphasis on 1st & 3rd beat. Exp II: metronome cueing at 2/4, 4/4 meter, emphasis on 1st & 3rd beat. Rhythmic verbal cues by researcher (heel off, toe off…)	Significant enhancement in 6-min walking distance in both Exp I & II after receiving auditory cueing, as compared to Ct. Significant reduction in timed 25-foot walking time, modified fatigue impact scale in both Exp I & II after receiving auditory cueing, as compared to Ct.
Conklyn et al. (41)	Effect of rhythmic auditory cueing on gait in patients affected from multiple sclerosis	Exp: 3F, 2M (47 ± 10.5) Ct: 4F, 1M (50.2 ± 5.4)	5	Functional ambulation performance, double support percentage (right/left), cadence, stride length (right/left), gait velocity, step length (right & left), norm velocity & timed 25-foot walking test	Exp: Pre-test, gait performance for 20 min per day for 4 weeks with rhythmic auditory cueing increased by 10% of attained cadence on every evaluation of test, post-tests at week 1, week 2, week 3, week 6 Ct: same procedure but rhythmic auditory cueing only for 2 latter weeks	Rhythmic auditory cueing in music at +10% of preferred cadence on each evaluation post-test	Significant enhancement in cadence, stride length (right/left), gait velocity, step length (right & left), norm velocity after training with rhythmic auditory cueing for 1 week. Significant reduction in double support percentage (right/left) in Exp as compared to Ct.
Baram and Miller (42)	Effect of auditory on gait in patients affected from Multiple sclerosis	Exp: 10F, 4M (48.5 ± 8) Ct: 6F, 5M (25.4 ± 1.9)	4	Gait velocity, stride length, 10 m walking test	Pre-test, followed by rhythmic auditory cueing & 10 min follow-up short term residual performance test	Rhythmic auditory cueing modified in real-time with steps	Significant enhancement in gait speed & stride length with rhythmic auditory cueing. Significant enhancement in short-term residual performance with auditory cueing.

The inclusion criteria for the studies was (i) Performed studies were either randomized controlled trials, cluster randomized controlled trials, or controlled clinical trials; (ii) Studies reporting reliable and valid spatiotemporal gait parameters (iii) Studies including dynamic aspects of gait stability (iv) Studies qualified PEDro methodological quality scale (≥4 score); (v) Experiments conducted on human participants; (vi) Published in a peer-reviewed academic journals; (vii) Articles published in English, German and Korean languages.

### Quality and risk of bias assessment

The quality of the studies was assessed using the PEDro methodological quality scale (72). The scale consists of 11 items addressing external validity, internal validity, and interpretability and can detect potential bias with fair to good reliability (73), and validity (72). A blinded rating of the methodological quality of the studies was carried out by the primary reviewer. Ambiguous issues were discussed between the 1st (SG) and the 2nd (IG) reviewer and consensus were reached. Included studies were rated, and interpreted according to scoring of 9–10, 6–8, and 4–5 considered of “excellent,” “good,” and “fair” quality (74), respectively. Inadequate randomization, non-blinding of assessors, no intention to treat analysis and no measurement of compliance were considered as major threats to biasing (75).

### Data analysis

This systematic review also included a meta-analysis approach even with a few number of studies (76), with an aim to develop a better understanding of the incorporated interventions (77). The presence and lack of heterogeneity asserted the use of either random or fixed effect meta-analysis (78). A narrative synthesis of the findings structured around the intervention, population characteristics, methodological quality (Table 2) and the type of outcome are also provided. Likewise, summaries of intervention effects for each study were provided in a tabular form (Table 2). A meta-analysis was conducted between pooled studies using CMA (Comprehensive meta-analysis V 2.0, USA). Heterogeneity between the studies was assessed using *I*^2^ statistics. The data in this review was systematically distributed and for each available variable pooled, dichotomous data was analyzed and forest plots with 95% confidence intervals are reported. The effect sizes were adjusted and reported as Hedge's g (79). Thresholds for interpretation of effect sizes were as follows: a standard mean effect size of 0 means no change, mean effect size of 0.2 is considered as a *small* effect, 0.5 is considered as a *medium* effect and 0.8 as a *large* effect (80). Interpretation of heterogeneity via *I*^2^ statistics was that values from 0-0, 25, 75% were viewed to sustain negligible, moderate, and substantial heterogeneity, respectively. A significance level of 0.05 was adopted.

## Results

### Characteristics of included studies

**Figure 1:**
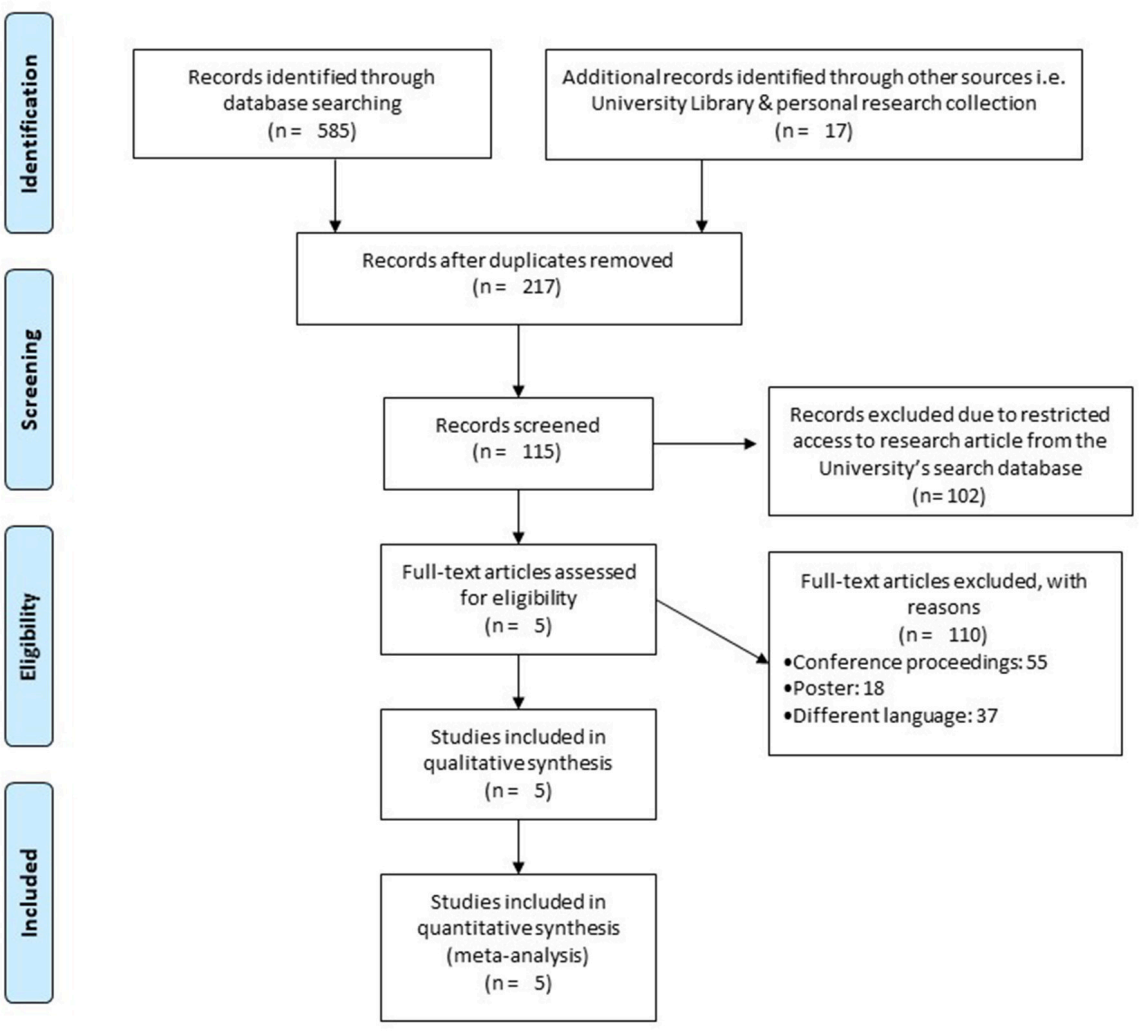
PRISMA flow chart for the inclusion of studies (68).

Our initial search yielded a total of 602 studies, which on implementing our inclusion/exclusion criteria, were reduced to five (Figure 1). Data from the included studies have been summarized in (Table 2). Of the five included studies, one was a randomized controlled trial, whereas four were controlled clinical trials.

#### Participants

A total of 188 participants were analyzed in the incorporated studies (144 females/40 males). All the studies evaluated a mixed gender sample size.

#### Risk of bias

**Figure 2:**
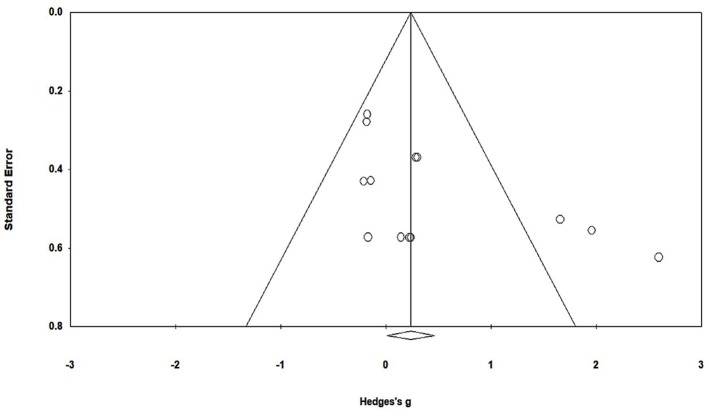
Funnel plot for Hedge's g & standardized effect for each effect in the meta-analysis. Each of the effect is represented in the plot as a circle. Funnel boundaries represent area where 95% of the effects are expected to abstain if there were no publication bias. The vertical line represents mean standardized effect of zero. Absence of publication bias is represented when the effects should be equally dispersed on either side of the line.

**Figure 3:**
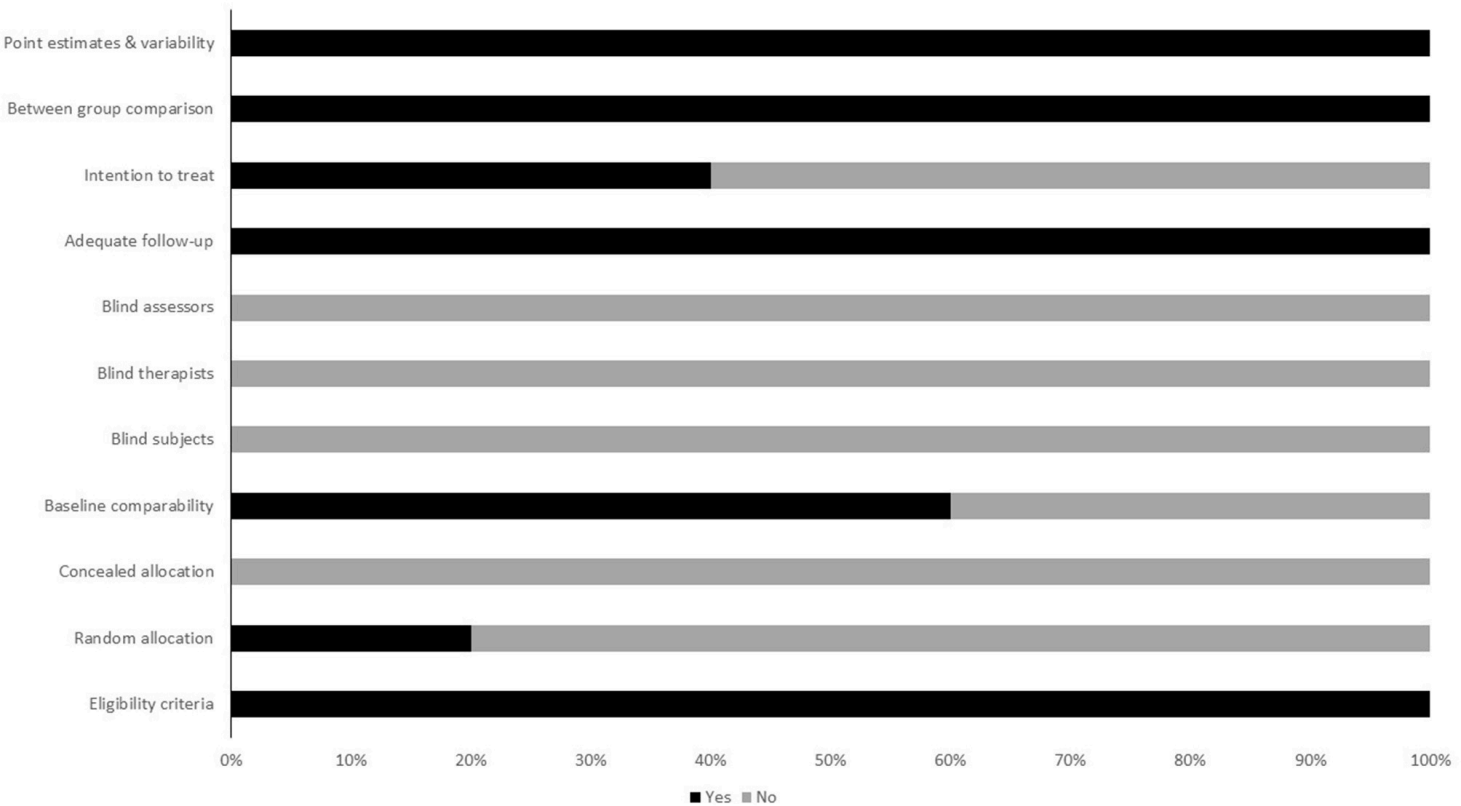
Risk of bias across studies.

To reduce the risks of bias, studies scoring ≥4 on PEDro were included in the review. Moreover, the limitation of research protocols to be included in the review were limited to gold standard randomized controlled trials, cluster randomized controlled trials and controlled clinical trials. The individual scores attained by the studies using the PEDro scale have been reported (Tables 2, 3). The average PEDro score for the five included studies were computed to be 5.2 out of 11, indicating fair-quality of the overall studies. One study scored 7, one scored 6, one scored 5, and two studies scored 4. Publication bias was analyzed by plotting a Hedge's g against standard error (Figure 2). Asymmetries concerning mean in the funnel plot might suggest bias (either positive or negative), in which case results are published. Risk of bias across the studies has been demonstrated in Figure 3.

**Table 3:** Individual Pedro scores for studies (1: point awarded, 0: no point awarded).

**Study**	**Pedro score**	**Point estimates & variability**	**Between group comparison**	**Intention to treat**	**Adequate follow-up**	**Blind assessors**	**Blind therapists**	**Blind subjects**	**Baseline comparability**	**Concealed allocation**	**Random allocation**	**Eligibility criteria**
Shahraki et al. 69	4	1	1	0	1	0	0	0	0	0	0	1
Seebacher et al. 70	7	1	1	0	1	0	0	0	1	1	1	1
Seebacher et al. 71	6	1	1	0	1	1	0	0	0	0	1	1
Conklyn et al. 41	5	1	1	0	1	1	0	0	0	0	0	1
Baram and Miller 42	4	1	1	0	1	0	0	0	0	0	0	1

### Meta-analysis

#### Outcomes

The results suggest evidence for a positive impact of rhythmic auditory cueing on spatiotemporal gait parameters patients affected from multiple sclerosis. In the five included studies, all the studies reported significant enhancements in gait parameters with application of rhythmic auditory cueing.

#### Meta-analyses

The evaluation of research studies via meta-analysis requires a strict inclusion criteria to efficiently limit the heterogeneity (81). However, among the pooled group of studies post a strict inclusion criterion, some amount of unexplained heterogeneity was still observed. Here, the few number of studies included in the meta-analysis limited our capability to perform additional sub-group analysis. The evaluated parameters were the spatiotemporal gait parameters such as gait velocity, cadence, stride length, and Timed-25 feet walking test.

#### Gait velocity (meter per second)

**Figure 4:**
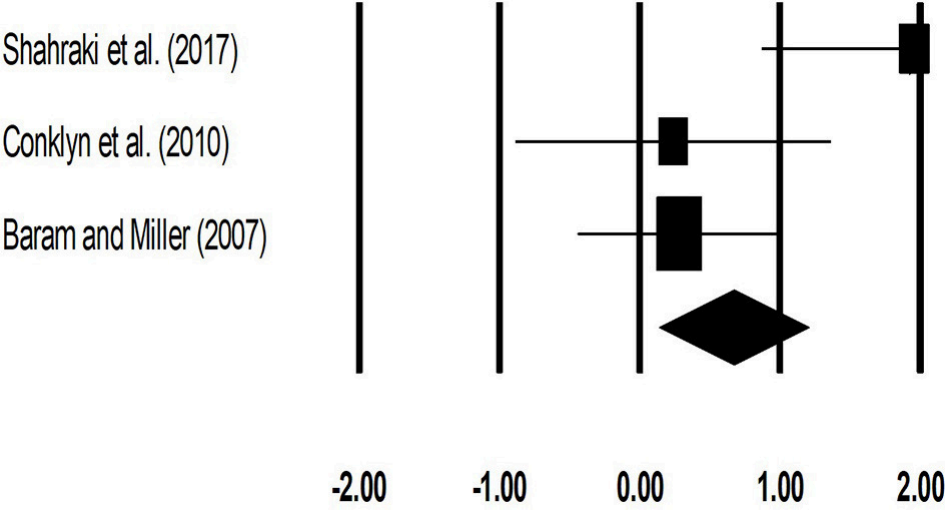
Forest plot illustrating individual studies evaluating the effects of rhythmic auditory cueing, on gait velocity (meter per second) for patients with multiple sclerosis. Weighted effect sizes; Hedge's g (boxes) and 95% C.I (whiskers) are presented, demonstrating repositioning errors for individual studies. The (Diamond) represents pooled effect sizes and 95% CI.

The meta-analysis on gait velocity for patients with multiple sclerosis revealed (Figure 4) a *medium* effect size in positive domain with moderate heterogeneity (Hedge's g: 0.67, 95% CI: 0.14 to 1.20, *I*^2^: 71.6%, *p* = 0.02).

#### Stride length (meters)

**Figure 5:**
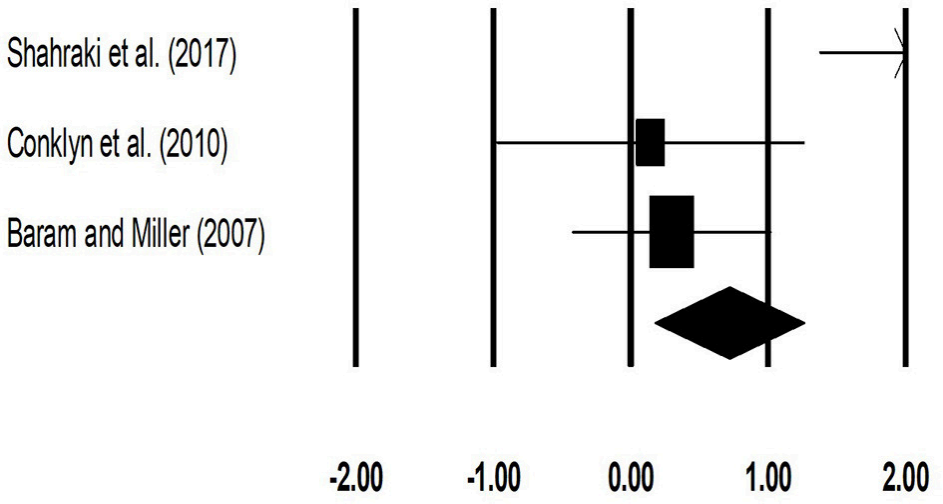
Forest plot illustrating individual studies evaluating the effects of rhythmic auditory cueing, on stride length (meters) for patients with multiple sclerosis. Weighted effect sizes; Hedge's g (boxes) and 95% C.I (whiskers) are presented, demonstrating repositioning errors for individual studies. The (Diamond) represents pooled effect sizes and 95% CI.

The meta-analysis on stride length for patients with multiple sclerosis revealed (Figure 5) a *medium* effect size in positive domain with substantial heterogeneity (Hedge's g: 0.71, 95% CI: 0.17 to 1.26, *I*^2^: 82.3%, *p* = 0.03).

#### Cadence (number of steps per minute)

**Figure 6:**
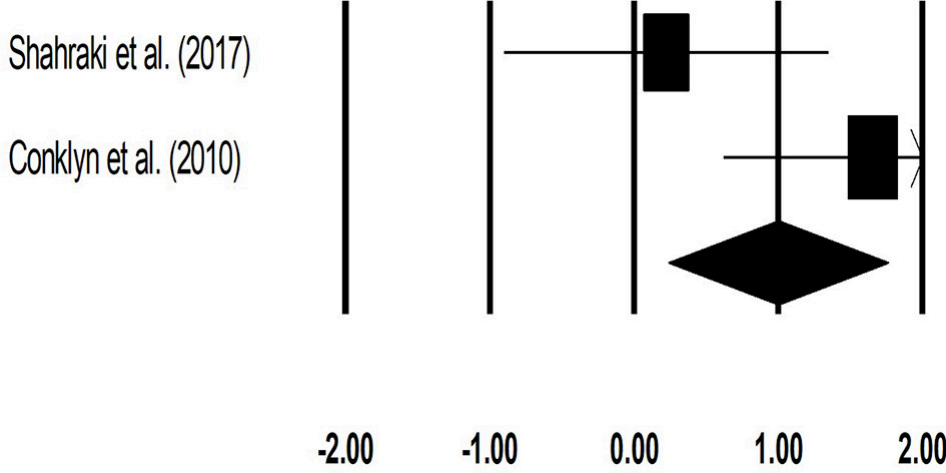
Forest plot illustrating individual studies evaluating the effects of rhythmic auditory cueing, on cadence (number of steps per minute) for patients with multiple sclerosis. Weighted effect sizes; Hedge's g (boxes) and 95% C.I (whiskers) are presented, demonstrating repositioning errors for individual studies. The (Diamond) represents pooled effect sizes and 95% CI.

The meta-analysis on cadence for patients with multiple sclerosis revealed (Figure 6) a *large* effect size in positive domain with substantial heterogeneity (Hedge's g: 1.00, 95% CI: 0.24 to 1.76, *I*^2^: 70.3%, *p* = 0.06).

#### Timed 25 feet walking test (seconds)

**Figure 7:**
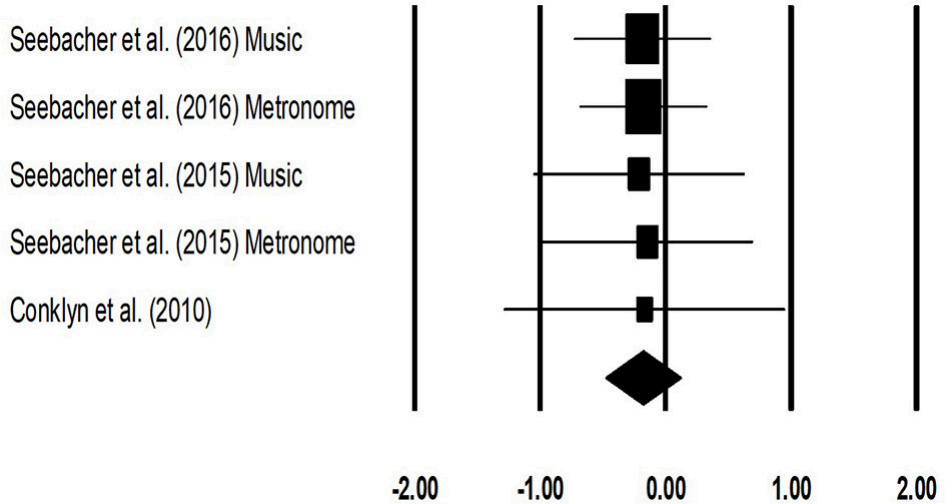
Forest plot illustrating individual studies evaluating the effects of rhythmic auditory cueing, on Timed 25 feet walking (seconds) test for patients with multiple sclerosis. Weighted effect sizes; Hedge's g (boxes) and 95% C.I (whiskers) are presented, demonstrating repositioning errors for individual studies. The (Diamond) represents pooled effect sizes and 95% CI.

The meta-analysis for timed-25 feet walking test for patients with multiple sclerosis revealed (Figure 7) a *small* effect size in negative domain with substantial heterogeneity (Hedge's g: −0.17, 95% CI: −0.48 to 0.12, *I*^2^: 0%, *p* > 0.05).

## Discussion

The primary objective of this present systematic review and meta-analysis was to develop a current state of knowledge for the effects of rhythmic auditory cueing on gait performance in patients with multiple sclerosis. All the included studies reported significant enhancements in gait performance post training with auditory cueing. The meta-analysis revealed significant small-to-large standardized effects for the beneficial influence of rhythmic auditory cueing on spatiotemporal gait parameters. Previous studies have reported a detrimental effect of multiple sclerosis on spatiotemporal gait parameters (16). For instance, Muratori et al. (82) has conclusively reported that a decrease in gait velocity, cadence, and stride length are important predictors for decreased quality of life, and increased fall related morbidity/mortality. Authors reported that gait velocity had a strong correlation with disease severity i.e., Expanded Disability Status scale and Multiple Sclerosis quality of life-54 scale. Likewise, Community Balance and Mobility scale has a strong relationship with step length and cadence (82). The current systematic review and meta-analysis reveals that training with rhythmic auditory cueing enhances gait velocity (Hedge's g: 0.67), stride length (0.70), cadence (1.0). Similarly, timed 25-foot walk test has been characterized as an important predictor to determine quality of life by focusing on functional independence and its impact on occupation, and social life (83–85). Here as well, a decrease in Timed 25-feet walking test (−0.17) was also reported in the analysis. This therefore suggests potential benefits of rhythmic auditory cueing for directly enhancing the quality of life and reducing morbidity/mortality ratios in patients with multiple sclerosis.

Neurophysiological mechanisms due to which auditory cueing enhances gait performance in patients with multiple sclerosis are not well-understood (16, 36, 42). In multiple sclerosis the onset of movement disorders is usually due to dysfunctions in white matter regions (16, 36, 86). Here inference can be drawn for the beneficial effects of auditory cueing, from a few studies analyzing the effects of auditory-sensorimotor training on white matter plasticity in musicians (87, 88). Bengtsson et al. (87) reported that auditory-sensorimotor training can increase myelination due to increased neural activity in the fiber tracts during training. The authors reported enhanced Fractional Anisotropy [usually reduced in multiple sclerosis (89, 90)] in corpus callosum, cortico-spinal, cortico-cortical tracts, and the posterior limb of the internal capsule. These neural structures are of critical importance when considering fine motor performance, bimanual coordination, auditory processing and motor learning (91, 92). Therefore, we hypothesize that training with auditory cueing could have enhanced the gait performance by facilitating the deficit white matter regions and/or mediating re-myelination. However, no research till date has analyzed the influence of auditory cueing on white matter plasticity in patients with multiple sclerosis. We strongly recommend future research to analyze the effects of auditory-motor entrainment on white matter plasticity in patients with multiple sclerosis.

Additionally, research in the past decades, for instance by Grimaud et al. (86) has reported that involvement of deep gray matter regions such as basal ganglia is unusual in patients with multiple sclerosis. However, recent evidence suggests that focal lesions and diffused neurodegeneration in deep gray matter regions such as basal ganglia, thalamus are an important precursors for contributing in development of neurological disabilities (1, 93–99), cognitive dysfunctions (97, 100), and the onset of fatigue (101, 102). Interestingly, research has also revealed a strong correlation between the quantitative susceptibility mapping of putamen and caudate nucleus with the severity of disease (97). Thereby suggesting greater involvement of gray matter structures with disease progression. This therefore again in our opinion might offer an additional explanation that application of rhythmic auditory cueing could have targeted the deficit basal ganglia circuitry similarly as in patients with Parkinson's disease to enhance gait performance, reduce the level of depression, anxiety, and fatigue in patients with multiple sclerosis [for a detailed mechanism see (47) and (27)]. Additionally, deficits in cerebellum [both gray and white matter regions (103)] have also been widely reported in patients with multiple sclerosis (104, 105). Here, findings of Molinari et al. (106) can justify the enhancements in gait performance with the application of auditory cueing. Molinari et al. (106) suggests that cerebellar dysfunctions such as in multiple sclerosis might impair the capability to consciously detect rhythmic variations for stabilizing motor response. However, the authors suggest that unconscious effects to entrain movements with external auditory cues might still be preserved in such patients. The authors suggest that in such cases the motor entrainment to auditory cueing might be induced unconsciously, independent of cerebellar processing at either the spinal or the cortical level. The authors proposed that computing of the timing information in such cases can be achieved peripherally i.e., directly in the auditory nerve by neural excitation patterns generated by precise physiological coding. This information can then be transferred directly into adjacent motor structures, which entrain with the neural motor codes and allow enhanced synchronization between the auditory stimuli and motor response (106, 107).

Furthermore, research suggests that application of auditory cueing can facilitate cortical reorganization in patients with multiple sclerosis (50). Till date only one research has analyzed the influence of rhythmic auditory cueing on cortical activation in patients with multiple sclerosis (108). The authors reported enhanced activation in left superior frontal gyrus, left anterior cingulate, and left superior temporal gyrus after gait training with rhythmic auditory cueing (36, 108). The increased activation in these neural centers has been associated with enhancements in executive functioning, auditory-motor entrainment, attention and motivation (50, 53). Similarly, enhanced activations in inferior colliculi (109), cerebellum, brainstem (110, 111), sensorimotor cortex (112, 113), premotor areas (114) have been reported post application of rhythmic auditory cueing in other movement disorders such as stroke and parkinsonism. Furthermore, modulation of neuromagnetic β oscillations (representing functional coordination between auditory-motor systems) with application of auditory cueing has been reported in auditory cortex, inferior frontal gyrus, somatosensory area, sensorimotor cortex and cerebellum (115). This ability of auditory cues has been recently demonstrated by Ross et al. (116) to facilitate immediate neural plasticity by facilitating feedforward mechanisms. Studies also suggest that training with rhythmic auditory cueing might offer reorganization of cortical and cerebellar circuits (63). Schaefer (117) for instance, suggested that auditory cueing infused with regularity and repetition of movement can result in an accelerated learning and neuroplasticity. Patients with multiple sclerosis have been reported to possess similar rapid-onset motor plasticity levels than that of healthy controls (118). Taken together, this evidence suggests strong therapeutic potential of external auditory stimulations to enhance gait performance in patients with multiple sclerosis. However, lack of conclusive evidence limits our interpretations, therefore we recommend future studies to analyse these components in neuroimaging studies.

Furthermore, extending beyond the neurophysiological effects of auditory stimulations Shahraki et al. (69) suggested that external auditory stimulations could also enhanced stability by facilitating the vestibular system via the medial-medial geniculate nuclei and organ of Corti (119). The authors demonstrated enhancements in spatiotemporal gait parameters with the application of rhythmic auditory cueing as compared to conventional physiotherapeutic gait training interventions in patients with multiple sclerosis. Likewise, Baram and Miller (42) too reported the beneficial aspects of external auditory cueing as compared to visual cueing. The authors reported higher gait velocity due to auditory cueing as compared to visual cueing, because of reduced reaction time facilitated by auditory stimulations during voluntary movements. The authors reported significant enhancements in gait velocity (Experimental: 12.8% vs. control: −3.0%) and stride length (8.3 vs. 0.3%) with the application of online rhythmic auditory cueing. Moreover, the authors demonstrated enhanced learning during residual performance (without auditory cueing) for both gait velocity (18.7 vs. 2.4%), and stride length (9.9 vs. 4.0%).

Moreover, we believe that the external auditory cueing could have also guided the gait of the patients' by explicitly synchronizing their ground contact and lift-off times (120). The cueing could have allowed the patients to effectively plan their movements before executing them (121). Likewise, enhanced kinematic efficiency and reduced variability in musculoskeletal activation patterns have been reported post training with rhythmic auditory cueing (26). Moreover, change in tempo of the auditory stimulation could have also played a major role in mediating gait performance. In the current review, only one study (69), trained their participants with a higher tempo (+10%) of rhythmic auditory cueing as compared to their preferred cadence. This “change in tempo” characteristic although not evaluated in the meta-analysis due to lack of data can serve as a crucial factor in rehabilitation of gait. For example, change in tempo has been associated with various neurophysiological changes such as increased neuronal activation in frontal-occipital cortical networks (122), and increased excitability of spinal motor neurons through the reticulospinal pathways (integral for reducing the response time in a motor task). Moreover, it has been reported that prolonged training with a constant pattern of rhythm can decrease fractal scaling of stride times from healthy 1/f structure (123–125). Here, we hypothesize that changing the tempo regularly during training can promote the development of a stable, and adaptable gait pattern. In rehabilitation this might serve as a measure to teach patients on how to regulate gait when passing through different fall prone environments.

Another crucial aspect analyzed in the current review is the effects of auditory cueing induced mental imagery in patients multiple sclerosis (71, 70). Labriffe et al. (126) reported higher activations in primary sensorimotor cortex and secondary somatosensory cortex bilaterally during the imagination of gait. The authors further reported correlated activations in bilateral somatosensory area and right pre-somatosensory area during mental imagery of gait. This training regime seems plausible in patients with multiple sclerosis where physical fatigue is a major concern for medical practitioners (127). Seebacher et al. (70) in their randomized controlled trial, asked the patients to kinaesthetically imagine gait from the first-person perspective with music and metronome induced rhythmic auditory cueing (71). The authors reported that mental imagery, which is usually diminished in patients with multiple sclerosis can be facilitated with rhythmic auditory cueing. Further, their study revealed significant enhancements spatiotemporal gait parameters such as timed 25-foot walking test, and 6-min walking test with the application of metronome/music-cued motor imagery groups. Here, comparable enhancements during 6-min walking test in music-cued (512.6 m), and metronome-cued (533.9 m) groups as compared to control group (471.2 m) clearly demonstrates beneficial effects of training with auditory cueing for enhancing physiological performance i.e., reduced fatigue. Likewise, improvements in multiple sclerosis related quality of life, pain, physical and mental health related quality of life were larger both music/metronome-cued groups as compared to control group. We would like to suggest that the beneficial effects of mental imagery here can also be effectively incorporated in home-based interventions. For instance, physiological fatigue might force the patient to train less at home. However, in such cases the patients can be taught to imagine themselves performing gait, while also imagining auditory cues. Previous studies suggest that the retention of enhancements in rehabilitation is dependent on how much the patient follows the treatment protocol at home (27, 28, 128). Therefore, developing interventions which can be easily followed by patients at home are desired. One of the included studies incorporated a home-based training intervention with external auditory cueing (41). Conklyn et al. (41) utilized a simple mp3 player to deliver rhythmic auditory cueing for practizing gait as a home-based intervention. The authors reported enhancements in spatiotemporal gait parameters and found increased patient adherence to the treatment. This type of home-based intervention could possibly be beneficial for people lacking proper exposure to medical interventions in developing countries (129). For instance, patients lacking effective medical resources can utilize smartphone devices with metronome applications for example Walkmate (124), Listenmee (130), or imagine gait with external stimulations or even imagine gait with auditory stimulations (joint audio-motor imagery).

Finally, a quantitative assessment for analyzing specific training dosage could not be performed in this study because of the limited amount of data and substantial heterogeneity in between the studies. Nevertheless, four of the included studies used a training regime that lasted for more than 17 min per session and was performed for at least three times a week for more than 3 weeks (41, 69–71). Likewise, based on the current evidence of training dosage for other movement disorders this dosage seems viable., for instance suggested a dosage of 25–40 min/session, for 3–5 sessions per week for patients with Parkinson's disease. Moreover, according to the findings of Bangert and Altenmüller (131) this training dosage seems plausible. The authors investigated cortical activation patterns during an audio-motor task and reported auditory-sensorimotor EEG co-activity after at least 20 min of training. Bangert and Altenmüller (131) speculate that this time frame is crucial for sensitive auditory monitoring, forming associations with the auditory target image in the working memory, during motor execution. Therefore, we suggest future studies to design training regimes with external auditory stimulations with at least 20 min training sessions. A limitation of the present review is that a meta-analysis was performed on a limited number of studies. Although, the main aim for conducting a meta-analysis was to allow a better understanding of the effects of auditory cueing over different spatiotemporal gait parameters for medical practitioners, patients and future researchers. This, however, does not rule out the possibility of incurring a type II error. We strongly suggest the reader to carefully interpret the results, while also considering the qualitative description of include studies provided in this review.

This review for the first time synthesized the evidence for effects of training with rhythmic auditory on gait in patients with multiple sclerosis. Our results are consistent with the findings of review studies suggesting the beneficial effects of rhythmic auditory cueing in healthy population (28), and population groups with movement disorders such as parkinsonism (47), stroke (57), and cerebral palsy (58). In conclusion, this review and meta-analysis suggests the incorporation of rhythmic auditory cueing for enhancing gait performance in patients with multiple sclerosis.

## Future directions

Extending beyond the beneficial effects of conventional isosynchronous auditory cueing, we recommend future studies to analyse the effects of biologically variable auditory stimulations on gait performance in patients with multiple sclerosis. Due to excessive sensory loss higher than normal threshold for action relevant acoustic input might be beneficial for patients with multiple sclerosis (132). Therefore, using ecologically valid action related sounds (walking on gravel, snow) conveying spatio-temporal information can possibly enhance saliency of sensory information for patients with multiple sclerosis (133–136). Similarly, analyzing the effects of methods providing real-time auditory information could possess considerable benefits for enhancing gait performance as well. This type of feedback allows converting the movement parameters in real-time to sound (mapping with pitch, amplitude). Here, the aim is to enhance motor perception and performance by targeting areas associated with biological motion perception (59, 137, 55). have shown that the synchronization of cyclic movement patterns with real-time auditory feedback can reduces variability and increases consistency of movements when compared with isosynchoronous rhythmic stimulations (56). According to this feedback can enable the patients to identify their own movement amplitudes and compare their produced sound patterns with the sound of an auditory movement model, thereby creating a new auditory reference framework. This then can possibly allow a better comparison between instructed and intended movement while simultaneously amplifying the internal representation of movements (138). In summary, we recommend future studies to focus on mediating auditory signal characteristics (ecologically valid, online feedback) for developing an efficient auditory stimulation, which can allow widespread benefits for patients with multiple sclerosis in both psychophysiological domains.

We also suggest future research to analyse the combined effects of external auditory stimulations with music therapy, as it might yield additional benefits to curb deficits in cognitive and physiological domain. For instance, Thaut et al. (139) demonstrated that musical mnemonics can facilitate a stronger oscillatory network synchronization in prefrontal regions during a word learning task in patients with multiple sclerosis. The authors suggested that musical stimuli might allow a “deep encoding” during a learning task and might also sharpen the timings of neural dynamics in brain which are normally degraded by the demyelination process. The authors also reported that this enhancement in cognitive performance was correlated with higher EDSS scores (139). Thereby, indicating that patients in more severe disease stages also benefited from the music facilitated “deep learning” strategies (139, 140). Likewise, enhanced cortical reorganization and regeneration in areas associated with cognition have been reported post music therapy (141, 142). We strongly recommend future research to analyse these effects in patients with multiple sclerosis. Furthermore, beneficial effects of music therapy in patients with multiple sclerosis has also been reported on respiratory musculature (143, 144). Future studies can focus on developing experimental protocols that use rhythmic cueing during music to facilitate breathing while performing gait. This approach might allow simultaneous strengthening of respiratory musculature while performing physical activities. Finally, it is important to consider the important psychological support that music therapy can offer to the patients with multiple sclerosis by reducing anxiety, depression, improving mood, self-acceptance and motivation (145–147). Future studies can also focus on analyzing these psychological aspects during the training regimes as this might allow in development of a multifaceted rehabilitation approach focusing on psychophysiological recovery of patients with multiple sclerosis.

## Author contributions

SG conceptualized the study, carried out the systematic-review, statistical analysis, and wrote the paper. IG assisted in the systematic-review process and reviewed the manuscript.

### Conflict of interest statement

The authors declare that the research was conducted in the absence of any commercial or financial relationships that could be construed as a potential conflict of interest.
